# Tunable transport and optoelectronic properties of monolayer black phosphorus by grafting PdCl_2_ quantum dots†

**DOI:** 10.1039/c8ra07053a

**Published:** 2018-10-15

**Authors:** Cuicui Sun, Yuxiu Wang, Yingjie Jiang, Zhao-Di Yang, Guiling Zhang, Yangyang Hu

**Affiliations:** School of Materials Science and Engineering, College of Chemical and Environmental Engineering, Harbin University of Science and Technology Harbin 150080 China guiling-002@163.com

## Abstract

The electronic, transport, and optoelectronic properties of monolayer black phosphorus (MLBP) are much influenced by grafting PdCl_2_ groups, demonstrated here by using density functional theory (DFT) and non-equilibrium Green's function (NEGF) as well as the Keldysh Nonequilibrium Green's Functions (KNEGF) methods. We find that the PdCl_2_ groups prefer to locate over the furrow site of MLBP and form a planar quadridentate structure of 
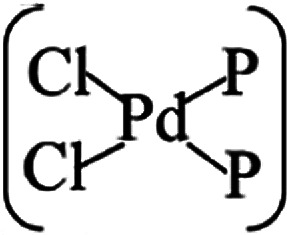
. The PdCl_2_ groups serve as quantum dots by introducing discrete flat levels between the MLBP valence band and the Fermi level (*E*_f_). The conductivity is much lowered after attaching PdCl_2_ quantum dots, due to the fact that the scattering effect of PdCl_2_ plays a major role in the process of electron transporting. A threshold voltage is found for the functionalized system with a large density of PdCl_2_ quantum dots, a valuable clue for exploring current switches. However, no evident threshold voltage is found for the pure MLBP. Electrons permeate easier through the armchair direction compared with the zigzag either in the pure MLBP or in the functionalized composites. More importantly, grafting PdCl_2_ quantum dots is very beneficial for enhancing photoresponse. The values of photoresponse for the modified species are about 20 times higher than the free MLBP. A significant photoresponse anisotropy is observed for both MLBP and *n*PdCl_2_-BP (*n* = 1, 2, and 4), contrary to the conductivity, the zigzag direction shows much stronger photoresponse than the armchair. All of the aforementioned unique properties make these new two-dimensional (2D) MLBP based materials especially attractive for both electronic and optoelectronic devices.

## Abstract 2

The photoelectric properties of the coordination functionalized monolayer black phosphorus by PdCl_2_ quantum dots are revealed for the first time.

## Graphical abstract

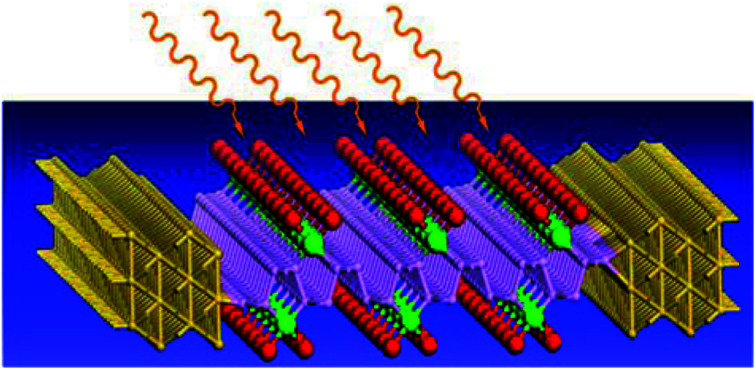

## 1. Introduction

Inspired by the great achievements of graphene, two-dimensional (2D) nanomaterials have received great attention in the fields of micro-/nano-electronics and optoelectronics due to their distinct confinement effect of electrons in two dimensions,^1^ strong in-plane covalent interactions,^2^ large lateral size,^3^ high exposure of surface,^4^ and so forth. After graphene with zero band gap^5^ and molybdenum disulphide with relatively low carrier mobility,^6^ monolayer black phosphorus (MLBP) has emerged as an important member of 2D materials family^7^ in recent years. In this layer, each P atom bonds to three neighboring P atoms through sp^3^ hybridized orbitals forming warped hexagons in a nonplanar fashion. To date, many production methods for MLBP have been developed, including liquid exfoliation,^8–12^ Ar^+^ plasma thinning process,^13^ photochemical etching,^14^ and mechanical exfoliation,^15,16^*etc.* Importantly, it has enormous potential to be used in high-performance electronic and optoelectronic devices because of its direct tunable band gap ranging from 0.3 to 2.0 eV,^17,18^ high carrier mobility of ∼1000 cm^2^ V^−1^ s^−1^ at room temperature,^7,17,19^ and anisotropic optical characteristics^20,21^ that could complement or exceed graphene and molybdenum disulphide in the next generation of novel devices.

In the electronic arrangement, the P atom with a valence shell configuration 3s^2^3p^3^ has five valence electrons available for bonding, and the existence of the lone pair makes MLBP reactive to air. Functionalization is proved to be an controllable and effective method to not only improve the stability but also to enrich the property^22,23^ of 2D materials. Tunability of electronic properties of 2D materials is crucial for their practical applications in electronics and optoelectronics. Up to now, many functionalized 2D black phosphorus (BP) have been reported, such as metal/BP contact systems,^24–27^ alkali metal/nonmetal atom doped BP,^28–31^ carbon nanotube/BP composites,^32^ as well as hBN/BP^33–35^ and MoS_2_/BP^36,37^ heterojunctions. Out of the above mentioned series, the most recent addition to the family are the functionalization of BP^38,39^ by adsorbing transition metal atoms on surface.^40–44^ It has been found that palladium (Pd) adatom adsorption on MLBP presents excellent electronic and optoelectronic behaviors.^41–43^ Up to now, research on functionalization of MLBP is predominantly focused on its physical modification; far less information is available regarding chemical modification, which is determined to be efficient in manipulating electronic and optoelectronic properties.^45^

In general, the Pd atom is a typical transition metal for coordinating P and Cl ligands.^46–48^ These complexes have shown wide spread applications in electronics, photoelectrochemistry, and electrochemistry.^49–51^ Usually, Pd has a 4d^10^ configuration and preferentially provides a dsp^2^ hybrid environment to coordinate with four ligands, *i.e.*, forms a planar quadridentate complex. The exposed P atoms of MLBP are perfect ligands by providing lone pair electrons to coordinate with the Pd atom of PdCl_2_ to form a planar quadridentate structure of 
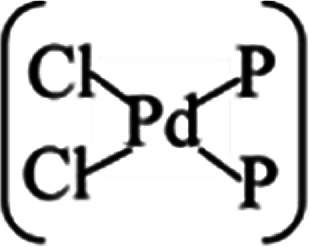
. Of particular interest in this paper is investigation of covalent functionalization of MLBP with PdCl_2_ groups including the effects of PdCl_2_ on electronic, transport, and optoelectronic properties.

## 2. Models and computational methods

**Fig. 1:**
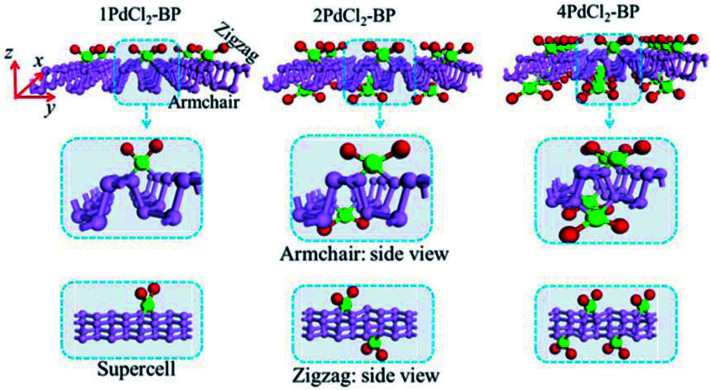
Optimized structures of three most stable systems of 1PdCl_2_-BP, 2PdCl_2_-BP, and 4PdCl_2_-BP.

For the periodic systems, we constructed the supercell as 2 × 4 (*x* × *y*) containing 32 P atoms (Fig. 1). We selected *n* PdCl_2_ groups (referred as *n*PdCl_2_, where *n* = 1, 2, and 4) to graft MLBP per supercell to study the effects of grafting density *n*. Hereafter, the functioned structures are denoted as *n*PdCl_2_-BP (*n* = 1, 2, and 4) for simplification. For the geometrical optimization, the PdCl_2_ was put at each adsorption site with all the atomic coordinates in the system being relaxed. The maximum force and maximum stress were set to the same value of 0.02 eV Å^−1^.

**Fig. 2:**
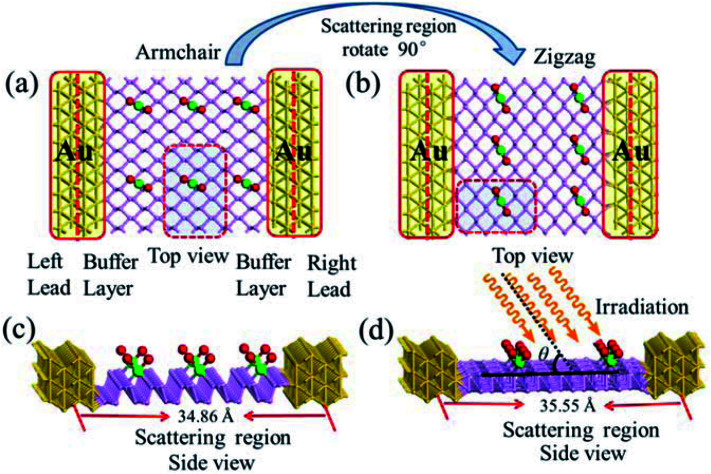
Exemplified two-probe devices by 1PdCl_2_-BP. (a) Top view of a-1PdCl_2_-BP; (b) top view of z-1PdCl_2_-BP; (c) side view of a-1PdCl_2_-BP; (d) side view of z-1PdCl_2_-BP. Irradiation on the scatter regions of two-probe devices are schematically shown in (d).

It has been reported that the MLBP displays anisotropic properties due to its anisotropic structure,^52,53^ which triggered us to calculate transport and optoelectronic properties for two mutually perpendicular directions, *i.e.*, the armchair and zigzag directions (Fig. 2(a and c)*vs.*2(b and d)). So we curved a nearly square plane (∼26.04 Å × 26.50 Å) of MLBP and *n*PdCl_2_-BP (*n* = 1, 2, and 4) as the scatter region to be sandwiched between two Au (100)-(9 × 3) electrodes to build two-probe devices. Such scatter region was formed by repeating each optimized supercell 3 times at the armchair direction and 2 times at the zigzag direction. When the scatter region used the zigzag edge to connect with the Au electrodes, the armchair direction properties were calculated (denoted as a-MLBP and a-*n*PdCl_2_-BP (*n* = 1, 2, and 4)). Another was just the reverse (nominated as z-MLBP and z-*n*PdCl_2_-BP (*n* = 1, 2, and 4)). As a benchmark test, the *E*_f_ positions of the two-probe systems with two or four buffer layers of Au were calculated at 0.0 V. We found that the *E*_f_ were almost locate at the same positions for the two cases (−3.533772 and −3.533858 eV for the two and four buffer layers, respectively). So we used two buffer layers of Au to perform further calculations. The nearest P–Au distance is 2.37 Å, corresponding to their covalent bonding.^54^ Transport current was computed by changing the applied bias voltage in the step of 0.2 V in the range of −1.0 to 1.0 V.

Electronic and transport property computations were performed by using the software package Atomistix ToolKit (ATK)^55–58^ based on a basis of the combination of density function theory (DFT) and non-equilibrium Green's function (NEGF) methods. The on-site correlation effect among 4d electrons of the Pd atom was accounted for by using the GGA+U scheme^58^ where the parameter U–J was set to be 6.0 based on the literature (6.0),^59^ which was also close to that (5.77) in the ATK_U database. The Perdew–Burke–Ernzerhof (PBE) exchange–correlation functional was employed. A single-ζ basis with polarization (SZP) was used for all atoms. A (7 × 5 × 1) *K*-point in the Brillouin zone (*x*, *y*, and *z* directions, respectively) was adopted, and 150 Ry cutoff energy was applied to describe the periodic wave function.

To gain an insight into the optoelectronic properties of MLBP and *n*PdCl_2_-BP (*n* = 1, 2, and 4), we irradiated the scatter regions of above mentioned two-probe systems with linearly polarized light (exemplified as Fig. 2(d)). The polarized angle *θ* was assigned between the scatter region plane and the irradiation direction. The photo energy was set from 0.0 to 1.0 eV with an interval of 0.1 eV. We calculated the photocurrent varying with *θ* from 0° to 180° at the 0.0 V bias voltage.

The optoelectronic properties of a-MLBP, z-MLBP, a-*n*PdCl_2_-BP, and z-*n*PdCl_2_-BP (*n* = 1, 2, and 4) were evaluated using the Nanodcal^60^ package which carried out DFT within the Keldysh Nonequilibrium Green's Functions (KNEGF).^60,61^ Standard norm-conserving nonlocal pseudopotentials were adopted to define the atomic cores, and SZP linear combination of atomic orbital basis set was used to expand physical quantities. The exchange–correlation potential was treated at the GGA+U with PBE.

## 3. Results and discussions

### 3.1 Stability and geometry

The MLBP is not residing in a flatland, instead, it forms a puckered hexagonal structure. The optimized supercell vectors of MLBP and *n*PdCl_2_-BP (*n* = 1, 2, and 4) were supplied in Table S1 in the ESI.† On the surface of this supercell one, two, or four PdCl_2_, corresponding to *n* = 1, 2, or 4, respectively, are considered to coordinate with the P atom for investigating the effect of grafting density. Taking into account all the circumstances, 20 configurations are computed for *n*PdCl_2_-BP (*n* = 1, 2, and 4), *i.e.*, 2 for 1PdCl_2_-BP, 10 for 2PdCl_2_-BP, and 8 for 4PdCl_2_-BP. Table S2 in the ESI† summarizes the optimized total energies of supercells of these 20 configurations. We find that the PdCl_2_ groups prefer to locate over the furrow site rather than upon the ridge position of the MLBP as the former has lower total energy than the latter. A planar structure of 
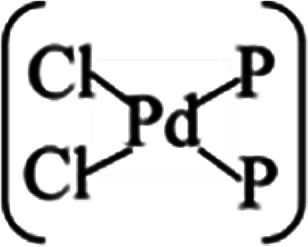
 is formed in these *n*PdCl_2_-BP complexes, a typical quadridentate structure for Pd. As for 2PdCl_2_-BP and 4PdCl_2_-BP, the PdCl_2_ components tend to evenly and staggerly distributed on both sides of MLBP. Finally, we chose three most stable configurations for *n* = 1, 2, and 4, respectively, as given in Fig. 1, to further investigate the electronic, transport, and optoelectronic properties.

For the three most stable structures of 1PdCl_2_-BP, 2PdCl_2_-BP, and 4PdCl_2_-BP, we have calculated the binding energies, *E*_b_, by using *E*_b_ = *E*_*n*PdCl_2_-BP_ − *E*_MLBP_ − *nE*_PdCl_2__, where *E*_*n*PdCl_2_-BP_, *E*_MLBP_, and *E*_PdCl_2__ denote the total energies of the functioned systems, pristine MLBP, and PdCl_2_ in the unit cell, respectively. For the PdCl_2_ calculation, the unit cell was set to be 30 × 30 × 30 Å, so that PdCl_2_ can be viewed as an isolated system. The calculated binding energies are all negative with increasing exoenergic values of −1.95, −3.83, and −7.35 eV from *n* = 1, 2 to 4 (Table 1), demonstrating that grafting PdCl_2_ groups to the MLBP surface is energetically favorable.

**Table 1:** Calculated binding energies *E*_b_ and geometries for MLBP and *n*PdCl_2_-BP (*n* = 1, 2, and 4)

Species	*E*_b_ (eV)	*r* ^in^_P–P_ (Å)	*r* ^out^_P–P_ (Å)	*r*_Pd–P_ (Å)	*r*_Pd–Cl_ (Å)	*D*_Pd–Pd_ (Å)	*D*_Cl–Cl_ (Å)
MLBP	—	2.224	2.244	—	—	—	—
1PdCl_2_-BP	−1.95	2.264	2.307	2.363	2.447	—	—
2PdCl_2_-BP	−3.83	2.268	2.345	2.364	2.442	6.359	7.667
4PdCl_2_-BP	−7.35	2.271	2.365	2.373	2.438	6.627	6.205

After optimizing, the P–P, Pd–P, Pd–Cl, and Pd–Pd bond lengths as well as the Cl–Cl distances for MLBP and *n*PdCl_2_-BP (*n* = 1, 2, and 4) are collected in Table 1. MLBP consists of double puckered planes; each atom is bound to two in-plane atoms with a bond length of *r*^in^_P–P_ = 2.224 Å and to one out-of-plane atom with a bond length of *r*^out^_P–P_ = 2.244 Å, fully in agreement with experimental observation.^62^ For *n*PdCl_2_-BP (*n* = 1, 2, and 4), the Pd–P bond lengths are *r*_Pd–P_ = 2.363–2.374 Å, in line with genral Pd–P chemical bond lengths calculated by M. A. Carvajal *et al.*^63^ (2.221–2.476 Å) and experimental data reported by V. I. Bakhmutov *et al.* (2.290–2.520 Å).^47^ This shows that the covalent bonds are formed between grafted PdCl_2_ groups and the P atoms. These P atoms (bonded to Pd) are pushed slightly inward (toward another plane), consequently, the *r*^in^_P–P_ and *r*^out^_P–P_ are elongated to 2.264–2.271 Å and 2.307–2.365 Å, respectively. The Pd–Cl bond lengths in *n*PdCl_2_-BP (*n* = 1, 2, and 4) are about *r*_Pd–Cl_ = 2.429–2.457 Å, in accordance with literature reported values of 2.333–2.575 Å.^63^ For *n*PdCl_2_-BP (*n* = 1, 2, and 4), the nearest Cl–Cl distances (*D*_Cl–Cl_) or Pd–Pd distances (*D*_Pd–Pd_) between two adjacent PdCl_2_ are all longer than 6.0 Å, implying negligible PdCl_2_–PdCl_2_ interactions in these considered systems.

### 3.2 Band structure

**Fig. 3:**
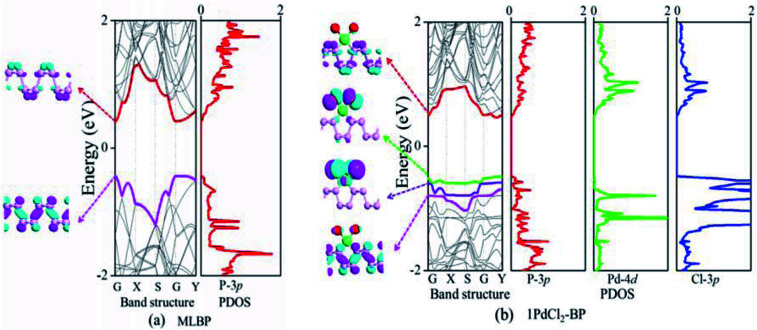
For (a) MLBP and (b) 1PdCl_2_-BP, computed band structures and PDOS as well as the Kohn–Sham orbital corresponding to the energy levels (highlighted in color lines) near *E*_f_ at the Γ point. The iso-surface value is 0.028 (e Å^−3^).

**Fig. 4:**
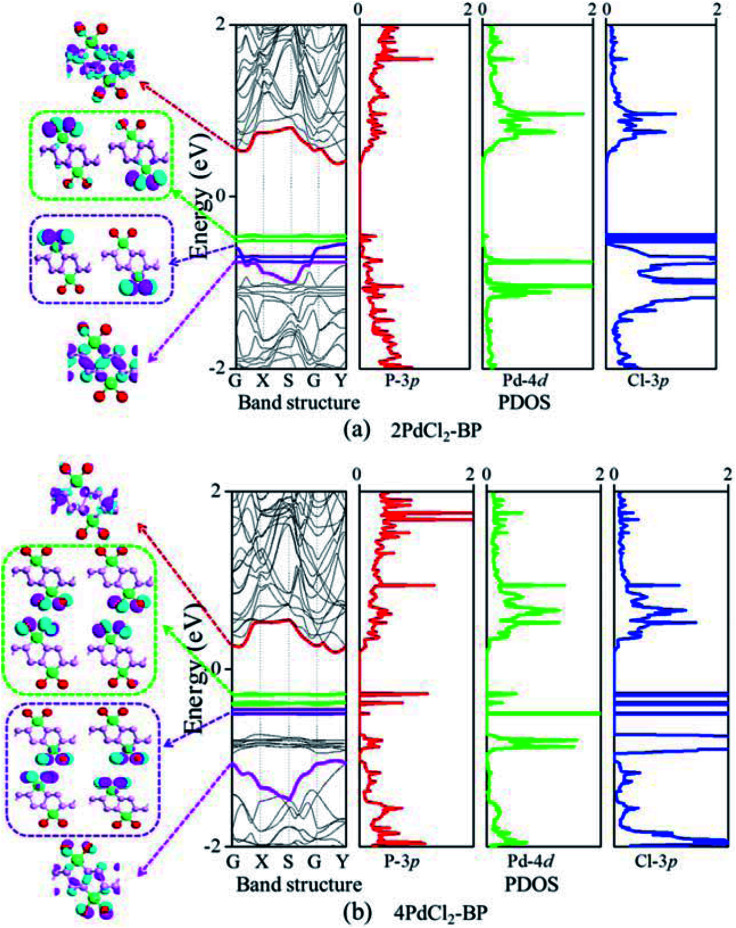
For (a) 2PdCl_2_-BP and (b) 4PdCl_2_-BP, computed band structures and PDOS as well as the Kohn–Sham orbital corresponding to the energy levels (highlighted in color lines) near *E*_f_ at the Γ point. The iso-surface value is 0.028 (e Å^−3^).

The band structure and projected density of states (PDOS) as well as the Kohn–Sham orbitals near the Fermi level (*E*_f_) of MLBP and *n*PdCl_2_-BP (*n* = 1, 2, and 4) were calculated as shown in Fig. 3 and 4. Clearly, the MLBP shows a semiconductive feature with a direct band gap (*E*_g_) of 0.85 eV, in agreement with the previous report value about 0.90 eV.^64–66^ Decoration of PdCl_2_ groups on MLBP surface can tune the transport property by altering the electronic structure. They may carry properties exhibited by each component as well as new properties generated as a result of their combination.

We can evidently find that *n*PdCl_2_-BP introduces flat PdCl_2_ bands just above the MLBP valence band and below the *E*_f_, indicating that the PdCl_2_ groups contribute localized states in the MLBP band gap region. The Kohn–Sham orbitals also clearly demonstrate the localized character of PdCl_2_ groups. The dispersion of some PdCl_2_ band structures around the Γ may be attributed to the large interaction between the Pd and Cl atoms. Correspondingly, PdCl_2_ groups give sharp PDOS peaks as localized valence states. In this sense, the anchored PdCl_2_ groups behave as quantum dots. These quantum dots mainly originate from the Cl-3p state as can be seen from its large PDOS. In general, quantum dots can form ionic like or covalent like interaction.^67^ In the former case, the electrons are localized on the individual dots which exhibit a weak tunnel coupling. For the latter, quantum dot electron states are quantum-mechanically coupled and show strong tunnel coupling. Clearly, *n*PdCl_2_-BP (*n* = 1, 2, and 4) systems bear spatially separated PdCl_2_ quantum dots with localized and discrete energy levels owing to the large separations between PdCl_2_ quantum dots, electron tunneling through direct coupling of PdCl_2_ is minor.

For a general planar quadridentate complex, the ligand field splitting of the Pd 4d electrons results in molecular orbitals being formed of principally a character of e_g_ (d_*yz*,*zx*_), a_1g_ (d_*z*^2^_), b_2g_ (d_*xy*_), and b_1g_ (d_*x*^2^–*y*^2^_). The former e_g_ (d_*yz*,*zx*_) and a_1g_ (d_*z*^2^_) are electron occupied leaving b_2g_ (d_*xy*_) and b_1g_ (d_*x*^2^–*y*^2^_) to be unoccupied. We can see from the PDOS, clearly, the e_g_ (d_*yz*,*zx*_) molecular orbitals locate just below the *E*_f_. Usually, the Pd quadridentate compounds belong to inner-orbital complexes with a zero magnetic moment. In *n*PdCl_2_-BP, the Cl_2_P_2_ ligands generate an uneven ligand field leading to a so-called Jahn–Teller effect. Pd forms a dsp^2^ hybrid environment to interact with the 3p orbitals of the P and Cl atoms. As a result, the degenerate orbitals of e_g_ (d_*yz*,*zx*_) are again split. Therefore, discrete bands appear in the MLBP band gap region of *n*PdCl_2_-BP (*n* = 1, 2, and 4).

**Fig. 5:**
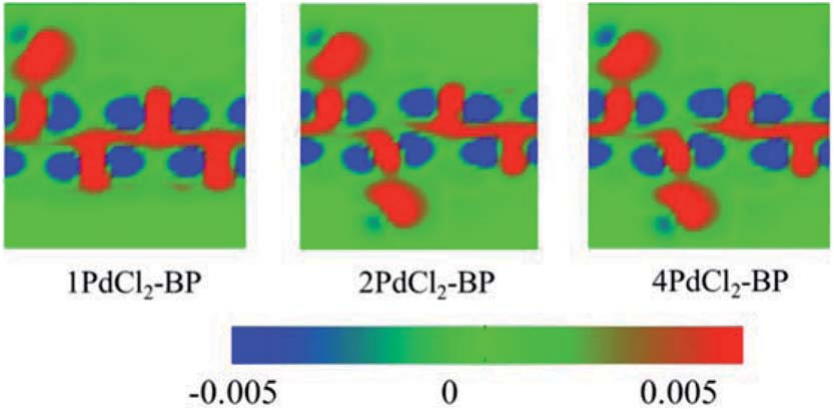
Computed electron difference densities of 1PdCl_2_-BP, 2PdCl_2_-BP, and 4PdCl_2_-BP. The green area means no electron transfer, red part represents obtaining electrons, while blue region indicates losing electrons.

To further investigate the interactions between MLBP and PdCl_2_, we calculated the electron difference densities for *n*PdCl_2_-BP (*n* = 1, 2, and 4) systems and the results are plotted in Fig. 5. The electron difference density refers to the difference between the self-consistent valence charge density and the superposition of atomic valence density, which indicates the coupling between the atoms in a certain system. The green area means no electron transfer, red part represents obtaining electrons, while blue region indicates losing electrons. Charge transfers from the MLBP substrate to the PdCl_2_ quantum dots can be observed from Fig. 5, suggesting a strong coupling between MLBP and PdCl_2_, which is an important factor in tuning the electronic and optoelectronic properties.

The *n*PdCl_2_-BP composites show p-type characteristics accompanied by the appearance of two kinds of midgap energy levels below the *E*_f_ (green and purple lines in Fig. 3 and 4). The higher one is close to the *E*_f_ and dominated by the Cl-3p state while the lower one is controlled by the Pd-4d state. The MLBP valence band is buried under the PdCl_2_ midgap energy levels and hybrids to some extent with Pd-4d/Cl-3p. Therefore, electrons can be transferred from the MLBP substrate to the PdCl_2_ quantum dots owing to the strong electronegativity of the Cl atom. The substrate MLBP and the appendant PdCl_2_ groups in *n*PdCl_2_-BP all contribute to the conduction band. The band gap values of MLBP in *n*PdCl_2_-BP do not bring much change relative to the pure MLBP, varying from 1.14 to 1.22 eV. However, the MLBP in *n*PdCl_2_-BP becomes to an indirect band gap semiconductor due to the orbital coupling of P with PdCl_2_, different from the direct feature in the pristine MLBP. In this case, the PdCl_2_ quantum dots play two-fold effects on the transport property. On one hand, they offer extra valence electrons to be excited to the conduction bands to participate in transporting which is beneficial for enhancing the conductivity. On the other hand, the electron excitation of PdCl_2_ midgap levels leaves holes for trapping electrons from MLBP, that is, PdCl_2_ quantum dots behave as additional scattering centers which degrade the mobility of the charge carriers. This is useful for devices which require fast switch off times.^68^ From the analysis above, it is concluded that the PdCl_2_ quantum dots exert an important effect in electron transporting, furthermore, such influence becomes more significant with the increasing grafting density *n*. This can be further confirmed by the calculated transport properties as addressed in the following section.

### 3.3 Transport property

To compute the electron transport properties of MLBP and *n*PdCl_2_-BP (*n* = 1, 2, and 4), the two-probe devices were constructed by sandwiching a curved 2D structure (26.05 Å × 26.51 Å) of MLBP and *n*PdCl_2_-BP (*n* = 1, 2, and 4) between two Au electrodes as shown by Fig. 2. The current *I* through the scatter region was calculated based on the formula (1):^57^1
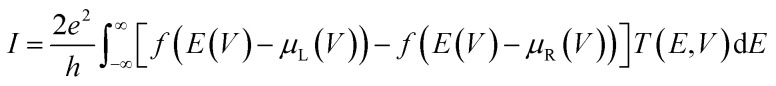
where *f* is the Fermi function; *μ*_L(R)_ is the chemical potentials of left (right) electrode; *T*(*E*,*V*) is the transmission function for electrons with energy *E* at certain bias *V*.

**Fig. 6:**
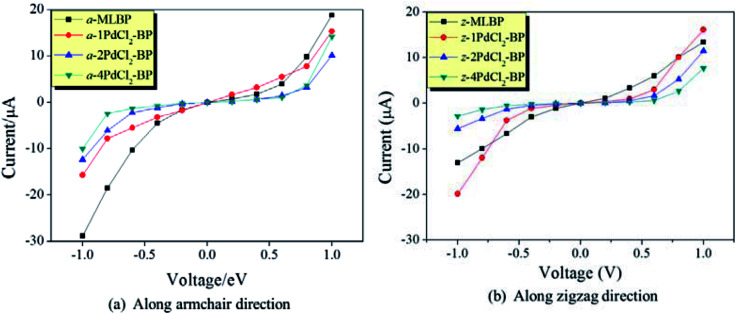
Computed *I*–*V* curves for MLBP and *n*PdCl_2_-BP (*n* = 1, 2, and 4) two-probe devices. (a) Transport along the armchair direction, and (b) transport along the zigzag direction.

The transmissions along the armchair (a-*n*PdCl_2_-BP) and zigzag (z-*n*PdCl_2_-BP) directions are both considered. The calculated current–voltage (*I*–*V*) curves are given in Fig. 6. As for MLBP, electrons permeate easier through the armchair direction compared with the zigzag. For instance, at −1.0 V bias voltage, the current magnitude of a-MLBP is −28.88 μA, while it goes down to −13.03 μA in the z-MLBP device. This anisotropic phenomenon is still preserved after being pinned with PdCl_2_ quantum dots. For example, under −1.0 V bias voltage, a-4PdCl_2_-BP gives a current of −10.01 μA while z-4PdCl_2_-BP presents a lower data of −2.82 μA. It is noteworthy that grafting PdCl_2_ quantum dots to the MLBP surface could significantly influence the transport properties. On one hand, the conductivity is much lowered after anchoring PdCl_2_ quantum dots, and the current magnitudes at a certain bias voltage follow the sequence of MLBP > 1PdCl_2_-BP > 2PdCl_2_-BP > 4PdCl_2_-BP, indicating that the scatter effect of PdCl_2_ plays a major role in the process of electron transporting. On the other hand, the threshold voltage becomes more and more distinct with the increasing grafting density *n*. Pure MLBP shows continuously rising current from 0.2 to 1.0 V bias voltage, no evident threshold voltage is found. Turn to 4PdCl_2_-BP, off-state is kept until the applied bias reaches to ±0.8 V, on-state begins at ±1.0 V bias voltage. Prospectively, grafting MLBP with a large density of PdCl_2_ quantum dots paves a new way toward achieving electronic switch devices.

**Fig. 7:**
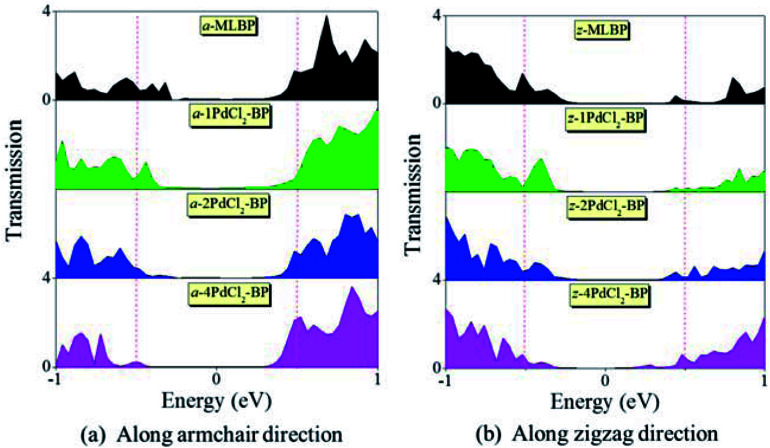
Transmission spectra of MLBP and *n*PdCl_2_-BP (*n* = 1, 2, and 4) two-probe devices at 1.0 V bias voltage. (a) Transport along the armchair direction, and (b) transport along the zigzag direction. Red dashed lines indicate the bias window.

**Fig. 8:**
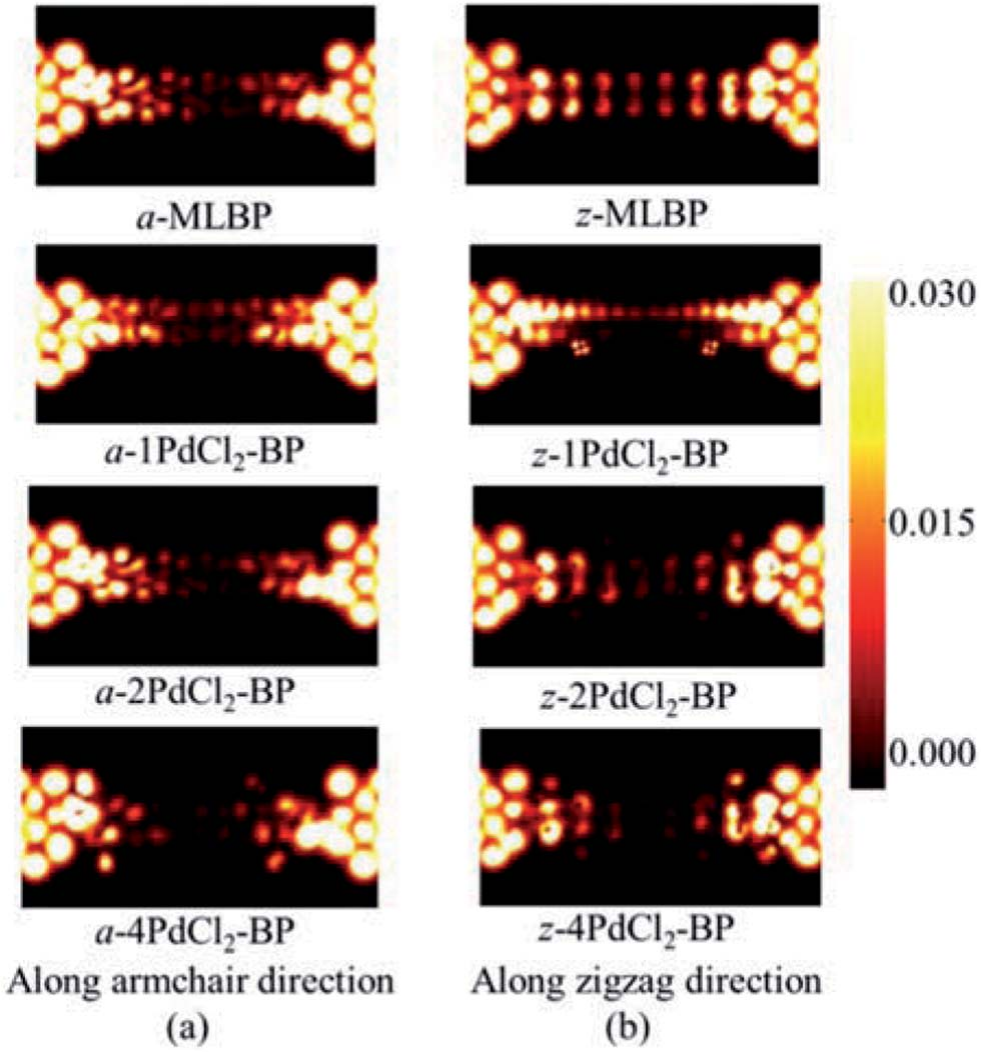
The computed LDOS at the Fermi level *E*_f_ for a-MLBP, z-MLBP, a-*n*PdCl_2_-BP, and z-*n*PdCl_2_-BP (*n* = 1, 2, and 4) two-probe devices at 1.0 V bias voltage.

**Fig. 9:**
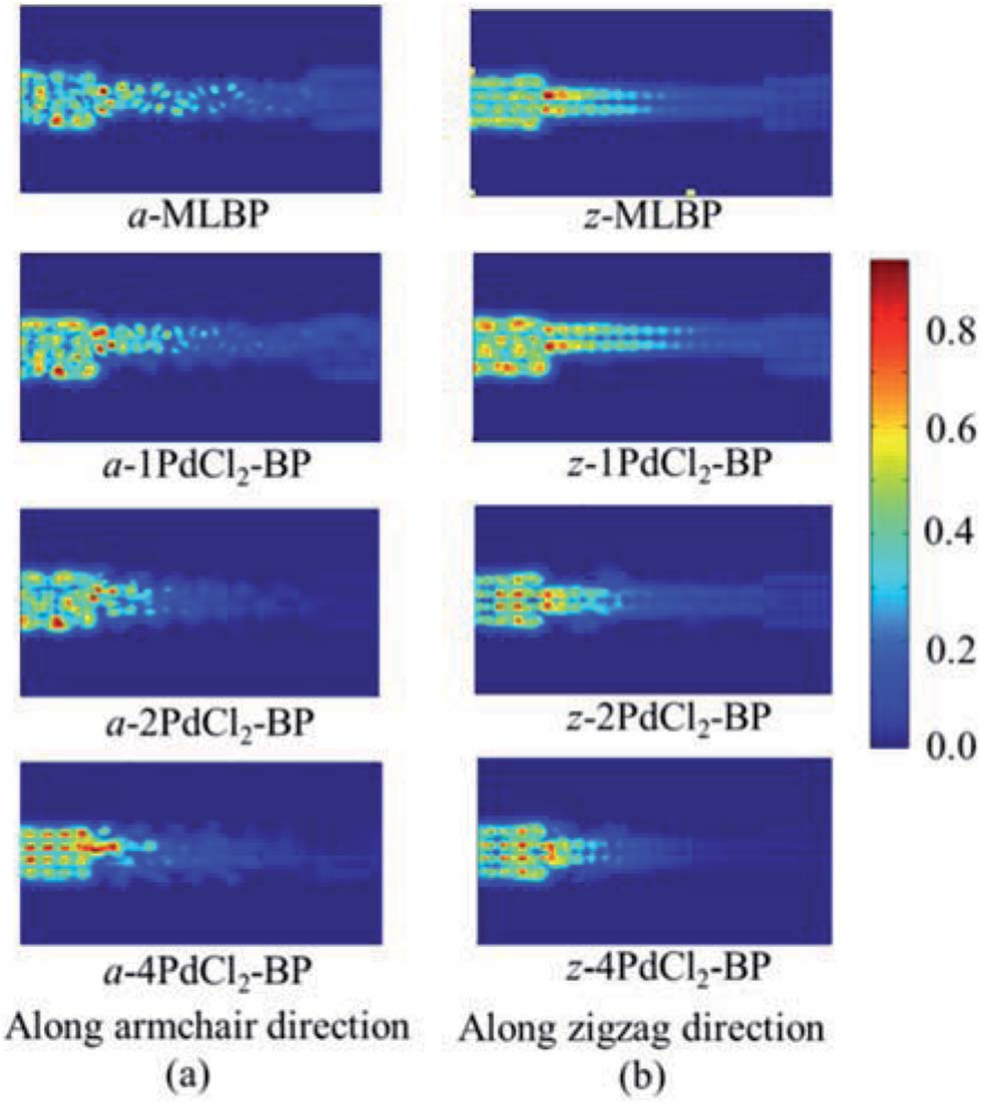
The real space scattering states at Fermi energy *E*_f_ of a-MLBP, z-MLBP, a-*n*PdCl_2_-BP, and z-*n*PdCl_2_-BP (*n* = 1, 2, and 4) two-probe devices.

To further shed light on effects of the PdCl_2_ grafting upon the transport properties, the transmission spectra (TS) for MLBP and *n*PdCl_2_-BP (*n* = 1, 2, and 4) at 1.0 V were calculated, as shown in Fig. 7. Usually, resonant peaks in the bias window contribute to the current. Here, the bias window refers to [–*V*/2, *V*/2]. Clearly, either for a-*n*PdCl_2_-BP device or for z-*n*PdCl_2_-BP device, the valence band resonant peaks (below the *E*_f_) in the bias window shrink gradually with the growing number *n*, again indicating the trap effect of PdCl_2_ quantum dots. Evidently, the conduction band resonant peaks (above the *E*_f_) in the bias window of a-*n*PdCl_2_-BP are much larger than those of z-*n*PdCl_2_-BP, and thereby the armchair direction gives a higher conductivity than the zigzag, in line with the *I*–*V* curves. These features can be further confirmed by the local density of state (LDOS) at the *E*_f_ (Fig. 8). Clearly, the LDOS of the scatter regions of both a-*n*PdCl_2_-BP and z-*n*PdCl_2_-BP fade away when adding more PdCl_2_ quantum dots, again indicating the block effect of PdCl_2_ quantum dots on electron transporting. Compared to a-*n*PdCl_2_-BP, the block phenomenon in z-*n*PdCl_2_-BP is more obvious, correlating well with the higher conductivity for the armchair direction as given by the *I*–*V* curves. We also calculated the real-space scattering states of the systems at the *E*_f_ for investigating the transport paths. Fourteen transport channels are obtained (*cf.* Fig. S1–S8 in ESI†), indicating that there exist fourteen subbands in the left electrode along the transport direction. Fig. 9 gives the most effective channels for MLBP and *n*PdCl_2_-BP (*n* = 1, 2, and 4). It can be seen that the transport channels tend to be closed if attaching more PdCl_2_ quantum dots due to their scattering effect, also intuitively explaining the reducing of the conductivity. Moreover, the penetrating channel of z-*n*PdCl_2_-BP is weaker at the right side than that of a-*n*PdCl_2_-BP, giving the evidence that charge carriers transport easily along the armchair direction related to the zigzag direction.

### 3.4 Optoelectronic property

Fig. 2 schematically shows the structures of our devices of *n*PdCl_2_-BP (*n* = 1, 2, and 4) for calculating optoelectronic properties along the armchair and zigzag directions. A linear polarized light was irradiated on the whole scattering region. The applied light has a polarization forming an angle *θ* with respect to the transport direction of the MLBP plane. The photoresponses were determined at different *θ* for photon energies ranging from 0 to 1.0 eV with an interval of 0.1 eV. The photocurrent *I*^(ph)^ can be obtained from the following formula (2):^69^2
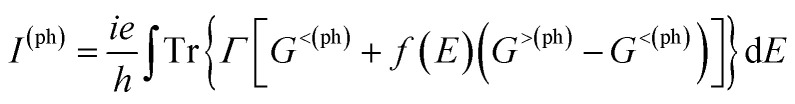


The photocurrent of eqn (2) can be separated into three terms:^70^3
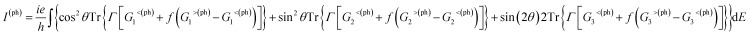
here *Γ* is the self-energy function, representing the coupling between the central scattering region and the left/right electrode; *G*^<(ph)^ is the lesser Green's function; *G*^>(ph)^ is the greater Green's function; *f*(*E*) is the Fermi–Dirac distribution function of the left and right electrode. It is easy to check that *I*^(ph)^ is proportional to the photon flux. Accordingly, the photoresponse function *R* can be define as4
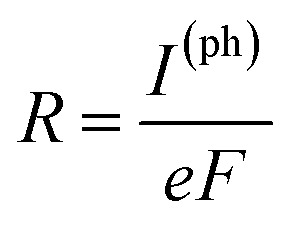
*versus* photon energy with different polarization direction of the light. Here *F* is the photon flux.

**Fig. 10:**
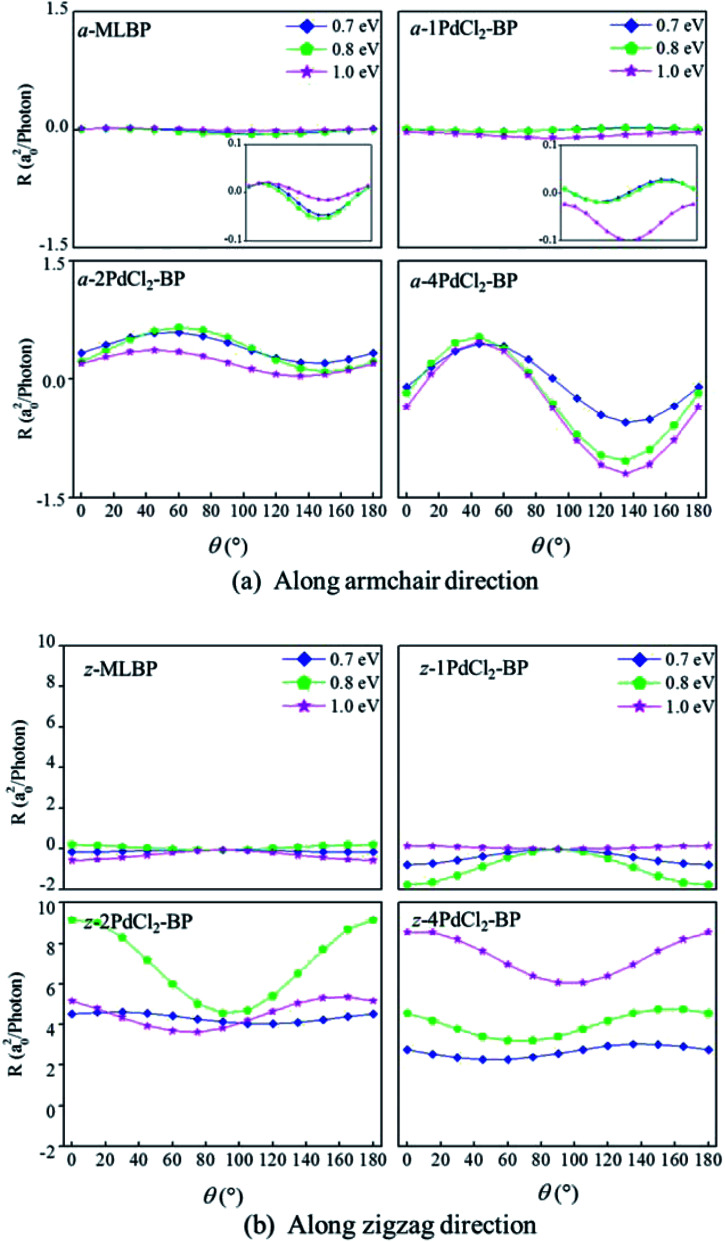
The calculated photoresponse *R* for MLBP and *n*PdCl_2_-BP (*n* = 1, 2, and 4) two-probe devices irradiated by linearly polarized light. (a) Photoresponse along the armchair direction, and (b) photoresponse along the zigzag direction.

**Fig. 11:**
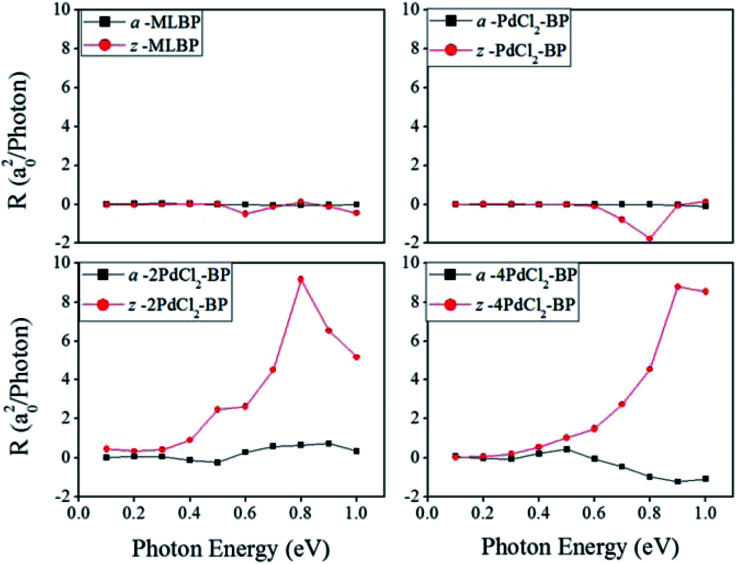
The calculated maximum photoresponse *R* for a-MLBP, z-MLBP, a-*n*PdCl_2_-BP, and z-*n*PdCl_2_-BP (*n* = 1, 2, and 4) two-probe devices irradiated by linearly polarized light.

Photoresponses along both armchair and zigzag directions of MLBP and *n*PdCl_2_-BP (*n* = 1, 2, and 4) at photo energies of 0.7, 0.8, and 1.0 eV are given in Fig. 10. According to formula (3), the photoresponses correlate directly to the sin^2^ *θ*, cos^2^ *θ*, and sin 2*θ* components. Therefore, the photoresponse curves in Fig. 10 take the sine or cosine shapes with *θ*, which are decided by complicated factors such as the geometric symmetry and band structures of the material. The sign of the photoresponse varies with *θ* and applied photon energy. This is due to the fact that the sign of the photoresponse is determined by the summation of all activated electrons with different velocity distributions. It is noteworthy that bonding PdCl_2_ quantum dots is much beneficial for enhancing photoresponses. Regardless of the direction, the maximum photoresponses within the considered photon energies are ordered as 4PdCl_2_-BP > 2PdCl_2_-BP > 1PdCl_2_-BP > MLBP. Values for a-4PdCl_2_-BP and z-4PdCl_2_-BP are up to 1.23 and 8.53 a_0_^2^/photon, respectively, 22 and 15 times larger than that of a-MLBP (0.0546 a_0_^2^/photon) and z-MLBP (0.579 a_0_^2^/photon). This is due to the fact that electrons can be excited easily by the light from the PdCl_2_ quantum dots which can be demonstrated from the band structures aforementioned. Fig. 11 shows the variation of maximum photoresponses with photo energies. Evidently, the *n*PdCl_2_-BP (*n* = 1, 2, and 4) nanostructures have produced photoresponse anisotropy: zigzag direction is about one order of magnitude larger than armchair. All of these fascinating photoresponse properties make these new 2D materials especially attractive for optoelectronic devices.

## 4. Summary

Electronic, transport, and optoelectronic properties of MLBP and *n*PdCl_2_-BP (*n* = 1, 2, and 4) are examined by using DFT and NEGF as well as the KNEGF methods. It is found that the PdCl_2_ quantum dots prefer to locate over the furrow site of MLBP and form a planar quadridentate structure of 
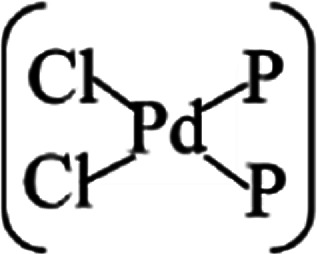
. A typical Pd–P coordinate bond is formed. The PdCl_2_ quantum dots introduce discrete flat levels just above the MLBP valence band and below the *E*_f_. The conductivity is much lowered by the PdCl_2_ grafting, due to the fact that the scatter effect of PdCl_2_ quantum dots play a major role in the process of electron transporting. For both parent MLBP and functioned *n*PdCl_2_-BP (*n* = 1, 2, and 4), the armchair direction shows higher conductivities than the zigzag. A threshold voltage is found for 4PdCl_2_-BP, a valuable clue for exploring current switches. More importantly, functionalization of PdCl_2_ quantum dots is much beneficial for enhancing photoresponse. Values of photoresponse for a-4PdCl_2_-BP and z-4PdCl_2_-BP are 22 and 15 times larger than that of a-MLBP and z-MLBP, respectively. A significant photoresponse anisotropy is found for both MLBP and *n*PdCl_2_-BP (*n* = 1, 2, and 4), contrary to the conductivity, the zigzag direction shows much stronger photoresponse than the armchair. All of the aforementioned unique properties make this new 2D MLBP based materials especially attractive for both electronic and optoelectronic devices.

## Conflicts of interest

There are no conflicts to declare.

## Supplementary Material

RA-008-C8RA07053A-s001

## References

[cit1] Geim A. K., Novoselov K. S. (2007). Nat. Mater..

[cit2] Fiori G., Bonaccorso F., Iannaccone G., Palacios T., Neumaier D., Seabaugh A., Banerjee S. K., Colombo L. (2014). Nat. Nanotechnol..

[cit3] Stoller M. D., Park S., Zhu Y., An J., Ruoff R. S. (2008). Nano Lett..

[cit4] Voiry D., Yang J., Chhowalla M. (2016). Adv. Mater..

[cit5] Castro Neto A. H., Guinea F., Peres N. M. R., Novoselov K. S., Geim A. K. (2009). Rev. Mod. Phys..

[cit6] Radisavljevic B., Radenovic A., Brivio J., Giacometti V., Kis A. (2011). Nat. Nanotechnol..

[cit7] Jing Y., Zhang X., Zhou Z. (2016). WIREs Computational Molecular Science.

[cit8] Yasaei P., Kumar B., Foroozan T., Wang C., Asadi M., Tuschel D., Indacochea J. E., Klie R. F., Salehi-Khojin A. (2015). Adv. Mater..

[cit9] Kang J., Wood J. D., Wells S. A., Lee J. H., Liu X., Chen K. S., Hersam M. C. (2015). ACS Nano.

[cit10] Zhang Y., Dong N., Tao H., Yan C., Huang J., Liu T., Robertson A. W., Texter J., Wang J., Sun Z. (2017). Chem. Mater..

[cit11] Zhao W., Xue Z., Wang J., Zhao X., Mu T. (2015). ACS Appl. Mater. Interfaces.

[cit12] Woomer A. H., Farnsworth T. W., Hu J., Wells R. A., Donley C. L., Warren S. C. (2015). ACS Nano.

[cit13] Lu W., Nan H., Hong J., Chen Y., Zhu C., Liang Z., Ma X., Ni Z., Jin C., Zhang Z. (2014). Nano Res..

[cit14] Kwon H., Seo S. W., Kim T. G., Lee E. S., Lanh P. T., Yang S., Ryu S., Kim J. W. (2016). ACS Nano.

[cit15] Fortin-Deschênes M., Levesque P. L., Martel R., Moutanabbir O. (2016). J. Phys. Chem. Lett..

[cit16] Chen Y., Jiang G., Chen S., Guo Z., Yu X., Zhao C., Zhang H., Bao Q., Wen S., Tang D., Fan D. (2015). Opt. Express.

[cit17] Li L., Yu Y., Ye G. J., Ge Q., Ou X., Wu H., Feng D., Chen X. H., Zhang Y. (2014). Nat. Nanotechnol..

[cit18] Das S., Zhang W., Demarteau M., Hoffmann A., Dubey M., Roelofs A. (2014). Nano Lett..

[cit19] Qiao J., Kong X., Hu Z. X., Yang F., Ji W. (2014). Nat. Commun..

[cit20] Xia F., Wang H., Jia Y. (2014). Nat. Commun..

[cit21] Castellanos-Gomez A. (2015). J. Phys. Chem. Lett..

[cit22] Kim J. S., Liu Y., Zhu W., Kim S., Wu D., Tao L., Dodabalapur A., Lai K., Akinwande D. (2015). Sci. Rep..

[cit23] Pei J., Xin G., Yang J., Wang X., Yu Z., Choi D. Y., Barry L. D., Lu Y. (2016). Nat. Commun..

[cit24] Yuchen D., Han L., Yexin D., Peide D. Y. (2014). ACS Nano.

[cit25] Li L., Engel M., Farmer D. B., Han S. J., Wong H. S. P. (2016). ACS Nano.

[cit26] Gong K., Zhang L., Ji W., Guo H. (2014). Phys. Rev. B: Condens. Matter Mater. Phys..

[cit27] Ma Y., Shen C., Zhang A., Chen L., Liu Y., Chen J., Liu Q., Li Z., Amer M. R., Nilges T., Abbas A. N., Zhou C. (2017). ACS Nano.

[cit28] Han C., Hu Z., Gomes L. C., Bao Y., Carvalho A., Tan S. J. R., Lei B., Xiang D., Wu J., Qi D., Wang L., Huo F., Huang W., Loh K. P., Chen W. (2017). Nano Lett..

[cit29] Li Q. F., Wan X. G., Duan C. G., Kuo J. L. (2014). J. Phys. D: Appl. Phys..

[cit30] Tan X. Y., Wang J. H., Zhu Y. Y., Zuo Y., Jin X. (2014). Acta Phys. Sin*.*.

[cit31] Zhao S., Kang W., Xue J. (2014). J. Mater. Chem. A.

[cit32] Xu G. L., Chen Z., Zhong G. M., Liu Y., Yang Y., Ma T., Ren Y., Zuo X., Wu X. H., Zhang X., Amine K. (2016). Nano Lett..

[cit33] Constantinescu G. C., Hine N. D. (2016). Nano Lett..

[cit34] Doganov R. A., Koenig S. P., Yeo Y., Watanabe K., Taniguchi T., Ozyilmaz B. (2015). Appl. Phys. Lett..

[cit35] Zhang P., Wang J., Duan X. M. (2016). Chin. Phys. B.

[cit36] Deng Y., Luo Z., J Conrad N., Liu H., Gong Y., Najmaei S., Ajayan P. M., Lou J., Xu X., Ye P. D. (2014). ACS Nano.

[cit37] Ye L., Li H., Chen Z., Xu J. (2016). ACS Photonics.

[cit38] Kumar V., Brent J. R., Shorie M., Kaur H., Chadha G., Thomas A. G., Lewis E. A., Rooney A. P., Nguyen L., Zhong X., Burke M. G., Haigh S. J., Walton A., Mcnaughter P. D., Tedstone A. A., Savjani N., Muryn C. A., O'Brien P., Ganguli A. K., Lewis D. J., Sabherwal P. (2016). ACS Appl. Mater. Interfaces.

[cit39] Li Q., Zhou Q., Niu X., Zhao Y., Chen Q., Wang J. (2016). J. Phys. Chem. Lett..

[cit40] Koenig S. P., Doganov R. A., Seixas L., Carvalho A., Tan J. Y., Watanabe K., Taniguchi T., Yakovlev N., Castro Neto A. H., Özyilmaz B. (2016). Nano Lett..

[cit41] Kulish V. V., Malyi O. I., Persson C., Wu P. (2015). Phys. Chem. Chem. Phys..

[cit42] Hu T., Hong J. (2015). J. Phys. Chem. C.

[cit43] Suvansinpan N., Hussain F., Zhang G., Chiu C. H., Cai Y., Zhang Y. W. (2016). Nanotechnology.

[cit44] Lei W., Zhang T., Liu P., Jose A. R., Liu G., Liu M. (2016). ACS Catal..

[cit45] Zhao Y., Wang H., Huang H., Xiao Q., Xu Y., Guo Z., Xie H., Shao J., Sun Z., Han W., Yu X. F., Li P., Chu P. K. (2016). Angew. Chem., Int. Ed..

[cit46] Alonso E., Forniés J., Fortuño C., Lledós A., Martín A., Nova A. (2009). Inorg. Chem..

[cit47] Bakhmutov V. I., Bozoglian F., Gómez K., González G., Grushin V. V., Macgregor S. A., Martin E., Miloserdov F. M., Novikov M. A., Panetier J. A., Romashov L. V. (2011). Organometallics.

[cit48] Berry D. E., Carrie P., Fawkes K. L., Rebner B., Xing Y. (2010). J. Chem. Educ..

[cit49] Moore L. R., Western E. C., Craciun R., Spruell J. M., Dixon D. A. (2008). Organometallics.

[cit50] Herbst K., Zanello P., Corsini M., Amelio N. D., Dahleburg L., Brorson M. (2003). Inorg. Chem..

[cit51] Folmer J. C. W., Turner J. A., Parkinson B. A. (1987). J. Solid State Chem..

[cit52] Tran V., Soklaski R., Liang Y., Yang L. (2014). Phys. Rev. B: Condens. Matter Mater. Phys..

[cit53] Du Y., Maassen J., Wu W., Luo Z., Xu X., Ye P. D. (2016). Nano Lett..

[cit54] Fei R., Yang L. (2014). Nano Lett..

[cit55] Perdew J. P., Burke K., Ernzerhof M. (1996). Phys. Rev. Lett..

[cit56] Brandbyge M., Mozos J. L., Ordejon P., Taylor J., Stokbro K. (2002). Phys. Rev. B: Condens. Matter Mater. Phys..

[cit57] Soler J. M., Artacho E., Gale J. D., Garcia A., Junquera J., Ordejon P., Sanchez-Portal D. (2002). J. Phys.: Condens. Matter.

[cit58] ATK, Version 13.8, atomistix a/s, 2013, www.quantumwise.com

[cit59] Gustafson S. Å., Dahlquist G. (2011). Chem. Phys. Lett..

[cit60] Taylor J., Guo H., Wang J. (2001). Phys. Rev. B: Condens. Matter Mater. Phys..

[cit61] Waldron D., Haney P., Larade B., MacDonald A., Guo H. (2006). Phys. Rev. Lett..

[cit62] Takahashi T., Tokailin H., Suzuki S., Shirotani I. (1985). J. Phys. C: Solid State Phys..

[cit63] Carvajal M. A., Miscione G. P., Novoa J. J., Bottoni A. (2005). Organometallics.

[cit64] Liu H., Neal A. T., Zhu Z., Luo Z., Xu X., Tomanek D., Ye P. D. (2014). ACS Nano.

[cit65] Das S., Zhang W., Demarteau M., Hoffmann A., Dubey M., Roelofs A. (2014). Nano Lett..

[cit66] Du Y., Ouyang C., Shi S., Lei M. (2010). J. Appl. Phys..

[cit67] Oosterkamp T. H., Fujisawa T., Wiel W. G. V. D., Ishibashi K., Hijman R. V., Tarucha S., Kouwenhoven L. P. (1998). Nature.

[cit68] Streetman B. G. and Banerjee S. K., Solid State Electronic Devices, Sixth Edition, Prentice Hall, 2006

[cit69] Zhang L., Gong K., Chen J., Liu L., Xiao D., Guo H. (2014). Phys. Rev. B: Condens. Matter Mater. Phys..

[cit70] Xie Y., Zhang L., Zhu Y., Liu L., Guo H. (2015). Nanotechnology.

