# Supplementary material for: Tunable transport and optoelectronic properties of monolayer black phosphorus by grafting PdCl2 quantum dots
Source: RSC Adv. 2018 Oct 15;8(61):35226–36. doi: 10.1039/c8ra07053a (PMC9087322; doi:10.1039/c8ra07053a)
Supplement: RA-008-C8RA07053A-s001 [file RA-008-C8RA07053A-s001.pdf]

Electronic Supplementary Material (ESI) for RSC Advances.
This journal is © The Royal Society of Chemistry 2018

Tunable transport and optoelectronic properties of monolayer black phosphorus by grafting $PdCl_2$ quantum dots

Cuicui Sun, Yuxiu Wang, Yingjie Jiang, Zhao-Di Yang, Guiling Zhang,* Yangyang Hu*

School of Materials Science and Engineering, College of Chemical and Environmental Engineering，Harbin University of Science and Technology, Harbin 150080, China.

Table S1 gives the optimized supercell vectors of MLBP and $n$$PdCl_2$-BP ($n$ = 1, 2, and 4).

Table S2 summarizes the optimized supercell total energies of 20 configurations for all $n$$PdCl_2$-BP ($n$ = 1, 2, and 4) systems.

Fig. S1-S8 show the real-space scattering states of the *a*-MLBP, *z*-MLBP, *a*-$n$$PdCl_2$-BP, and z-$n$$PdCl_2$-BP ($n$ = 1, 2, and 4) two-probe devices. Fourteen transport channels are obtained indicating that there exit fourteen subbands in the left electrode along the transport direction. It can be seen that the transport channels tend to be closed if more $PdCl_2$ quantum dots attached due to their scattering effect. Moreover, the penetrating channel of *z*-$n$$PdCl_2$-BP is weaker at the right side than that of *a*-$n$$PdCl_2$-BP, giving the evidence that charge carriers transport easily along the armchair direction related to the zigzag direction.

Additional result:

Table S1 The optimized supercell vectors of MLBP and nPdCl$_2$-BP (n = 1, 2, and 4).

Species	x (Å)	y (Å)	z (Å)
MLBP	8.714	13.253	30
1PdCl$_2$-BP	8.686	13.254	30
2PdCl$_2$-BP	8.426	13.231	30
4PdCl$_2$-BP	8.665	13.186	30

Table S2 The optimized supercell total energies of 20 configurations for all nPdCl$_2$-BP (n = 1, 2, and 4) systems.

1PdCl$_2$-BP	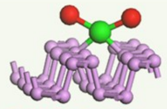 -10209.20 eV	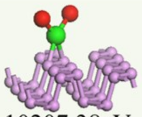 -10207.38eV			
2PdCl$_2$-BP	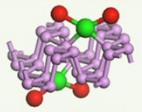 -11968.72 eV	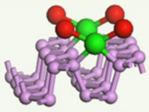 -11968.66 eV	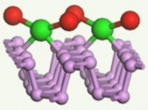 -11968.53 eV	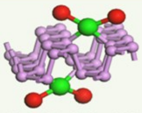 -11968.37 eV	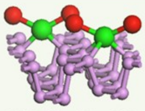 -11968.27 eV
	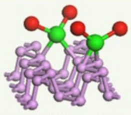 -11967.81 eV	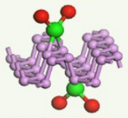 -11965.11 eV	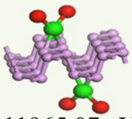 -11965.07 eV	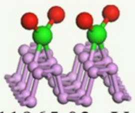 -11965.03 eV	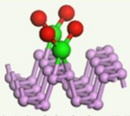 -11964.94 eV
4PdCl$_2$-BP	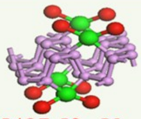 -15487.52 eV	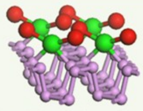 -15487.33 eV	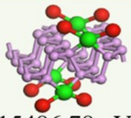 -15486.78 eV	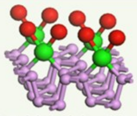 -15483.83 eV	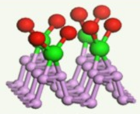 -15481.92 eV
	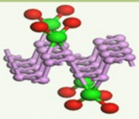 -15480.21 eV	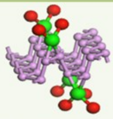 -15480.17 eV	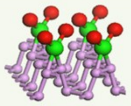 -15480.09 eV		

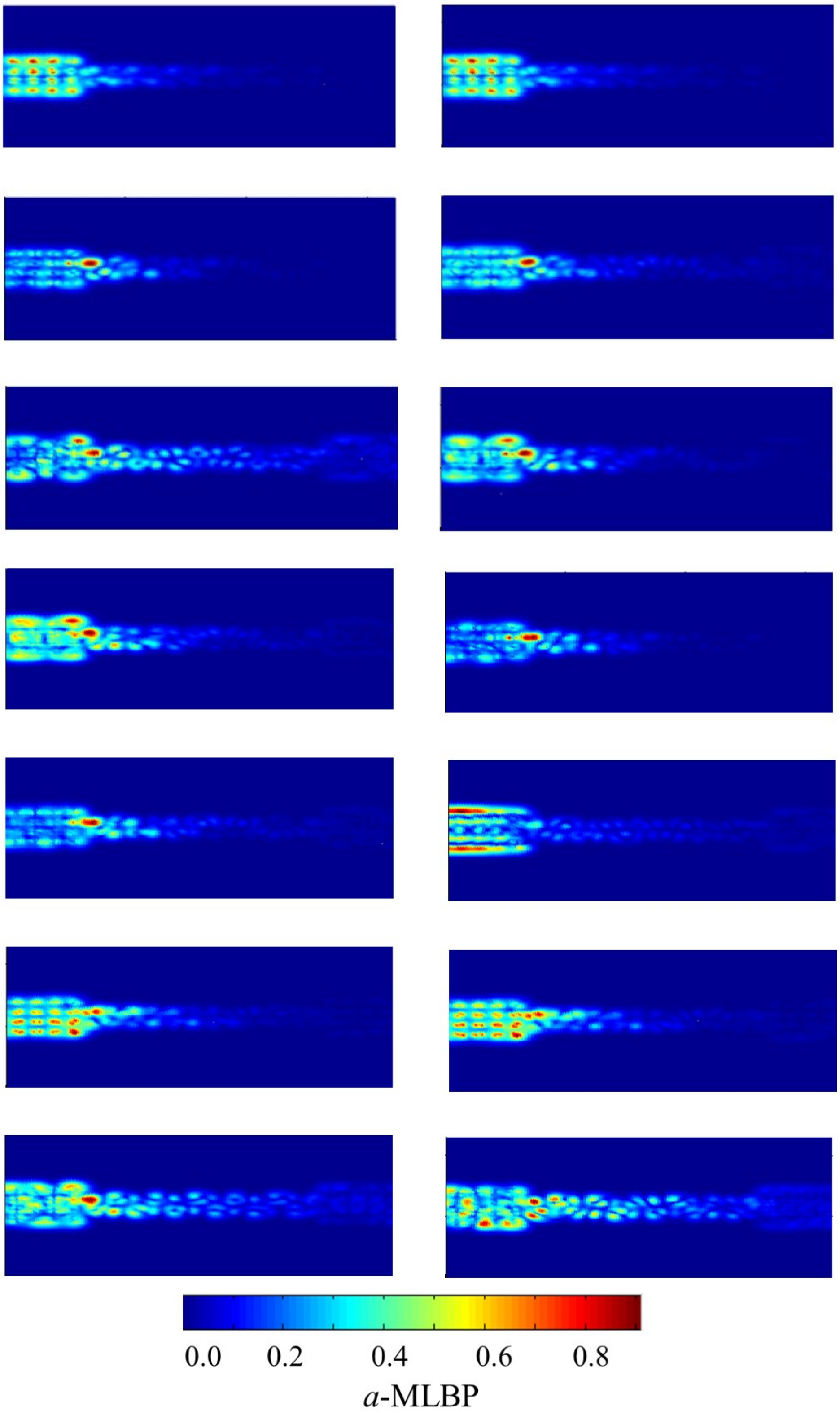

Fig. S1 The real space scattering states of *a*-MLBP at Fermi energy E_{f}.

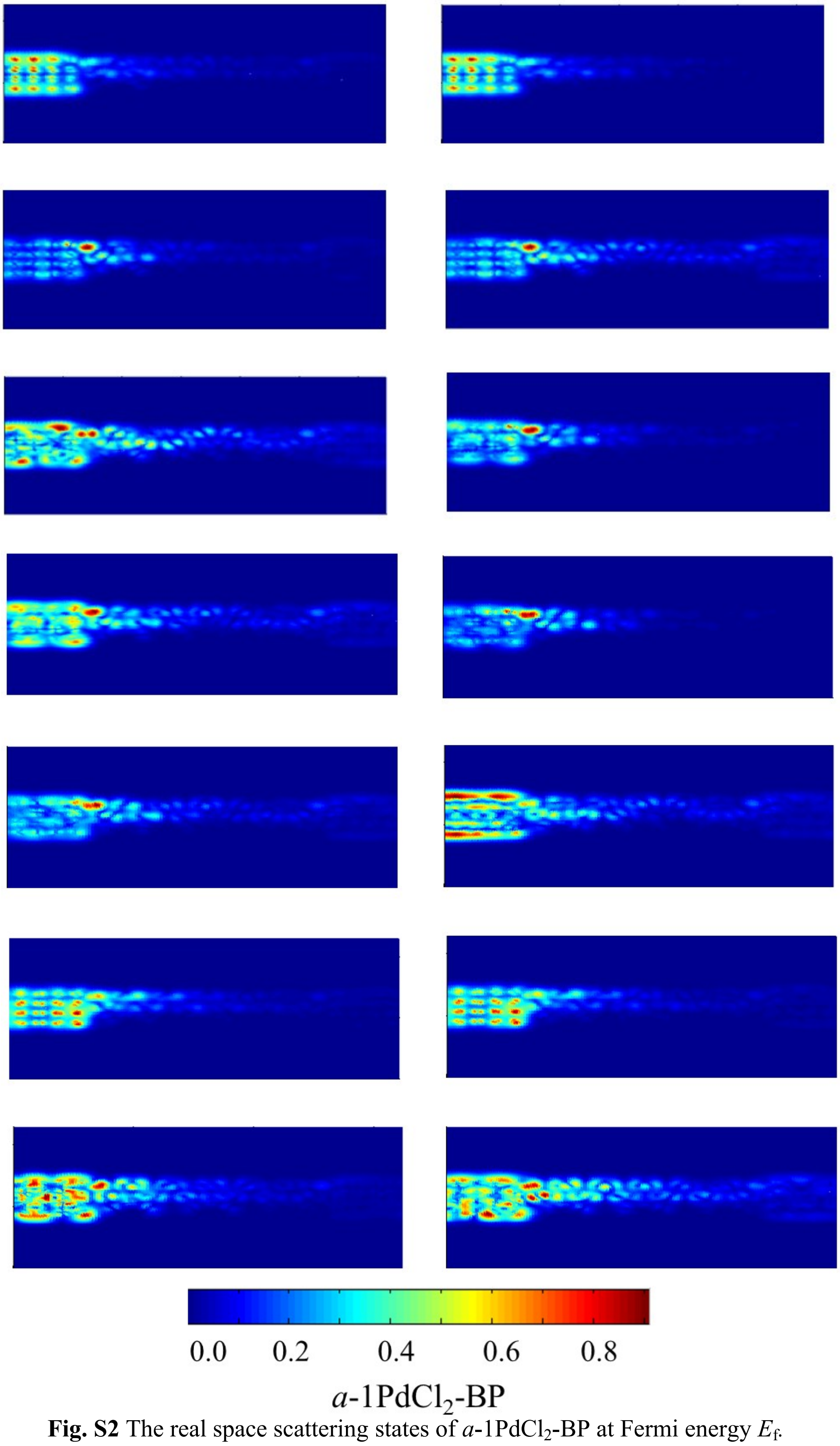

Fig. S2 The real space scattering states of a-1PdCl$_2$-BP at Fermi energy E_f.

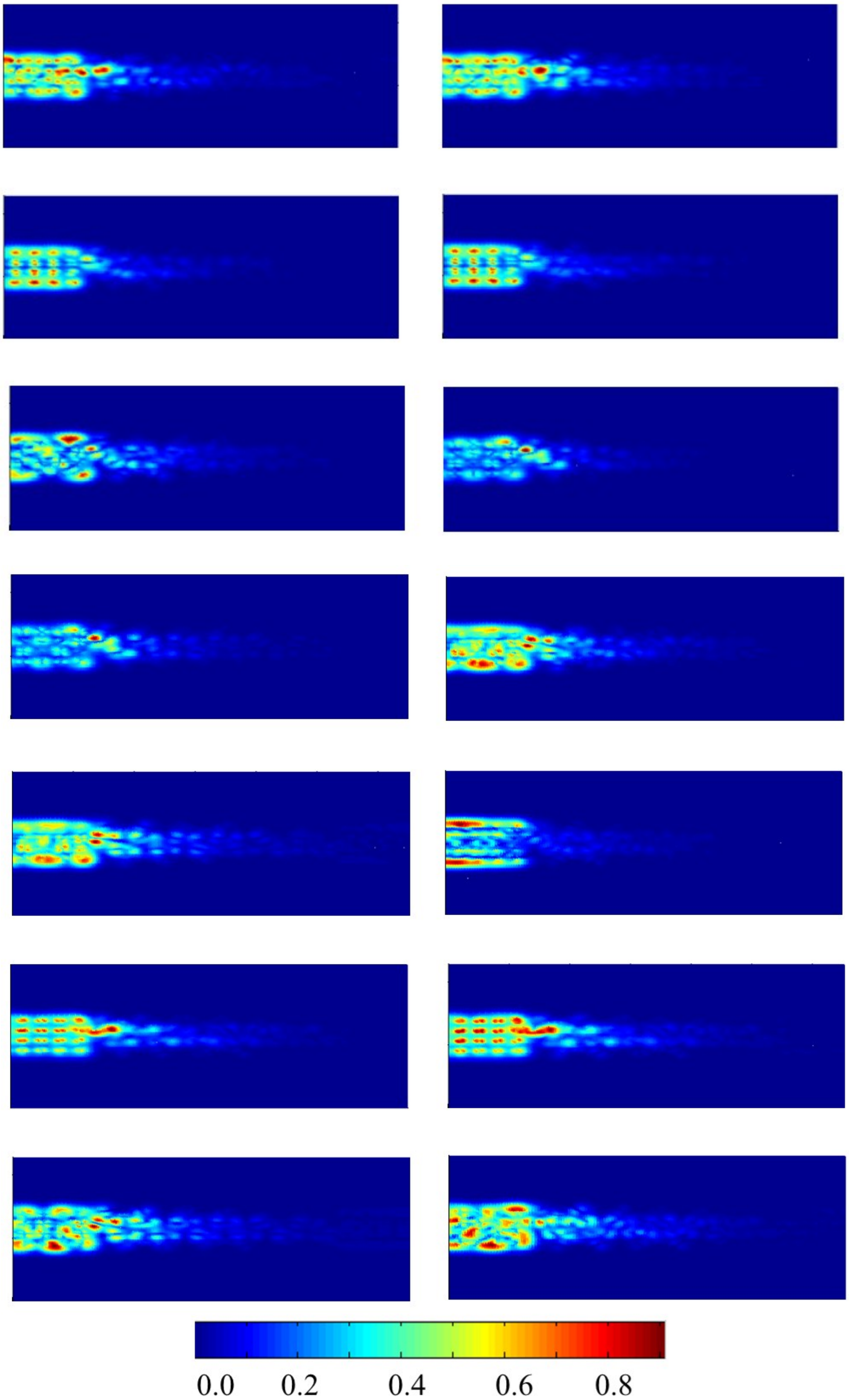

Fig. S3 The real space scattering states of a-2PdCl$_2$-BP at Fermi energy E_f.

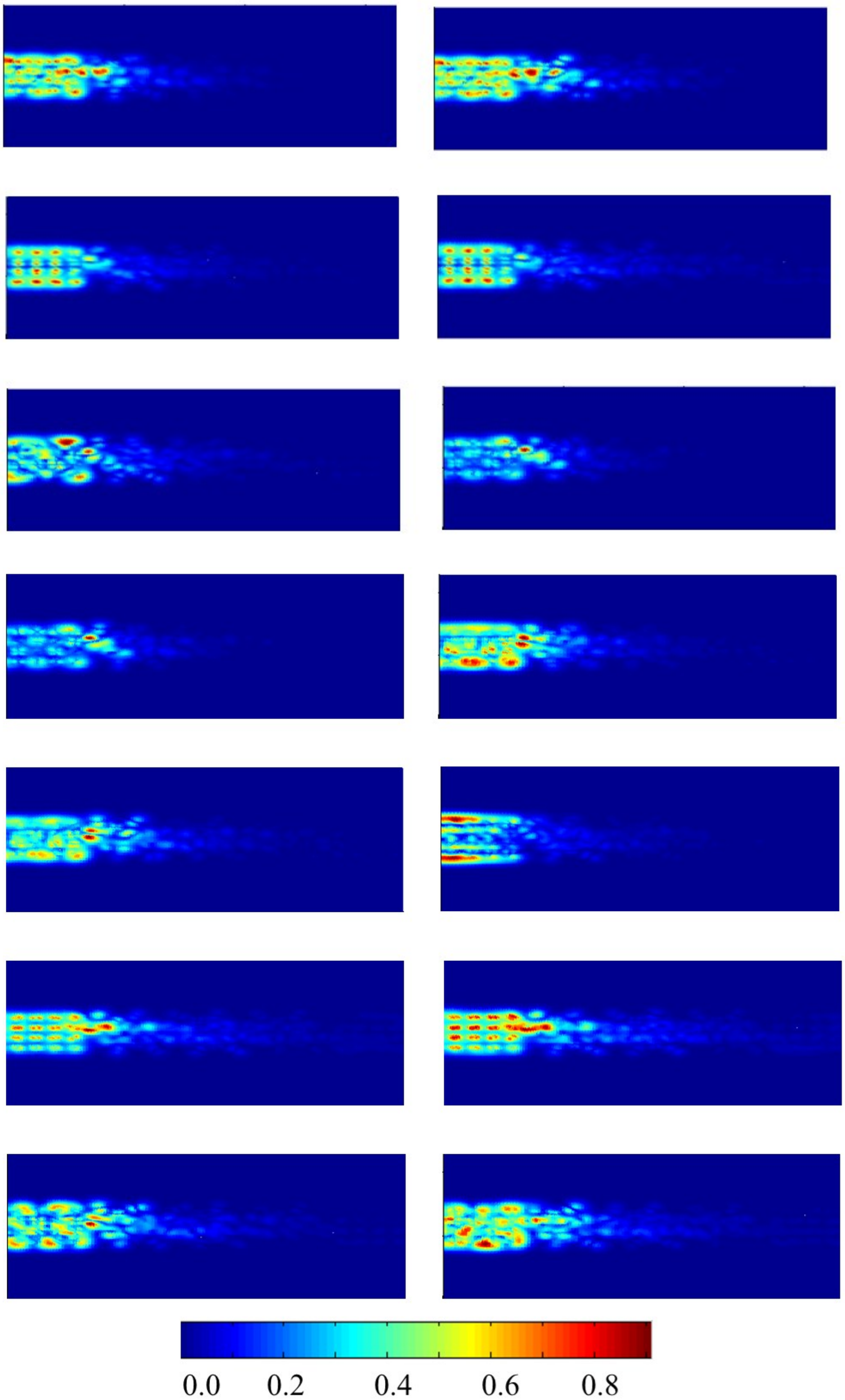

a-4PdCl$_2$-BP

Fig. S4 The real space scattering states of a-4PdCl$_2$-BP at Fermi energy E_f.

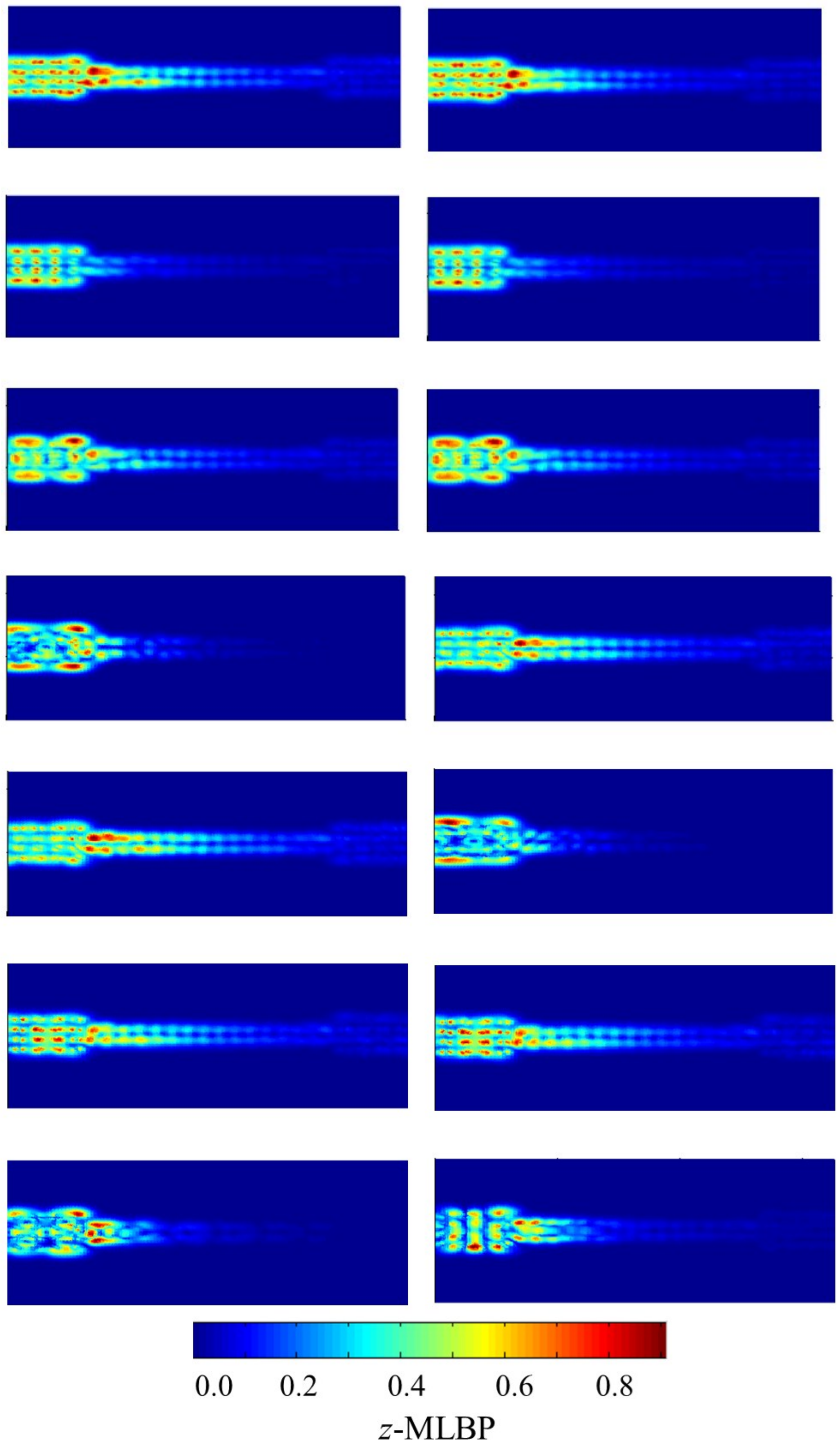

Fig. S5 The real space scattering states of *z*-MLBP at Fermi energy E_f.

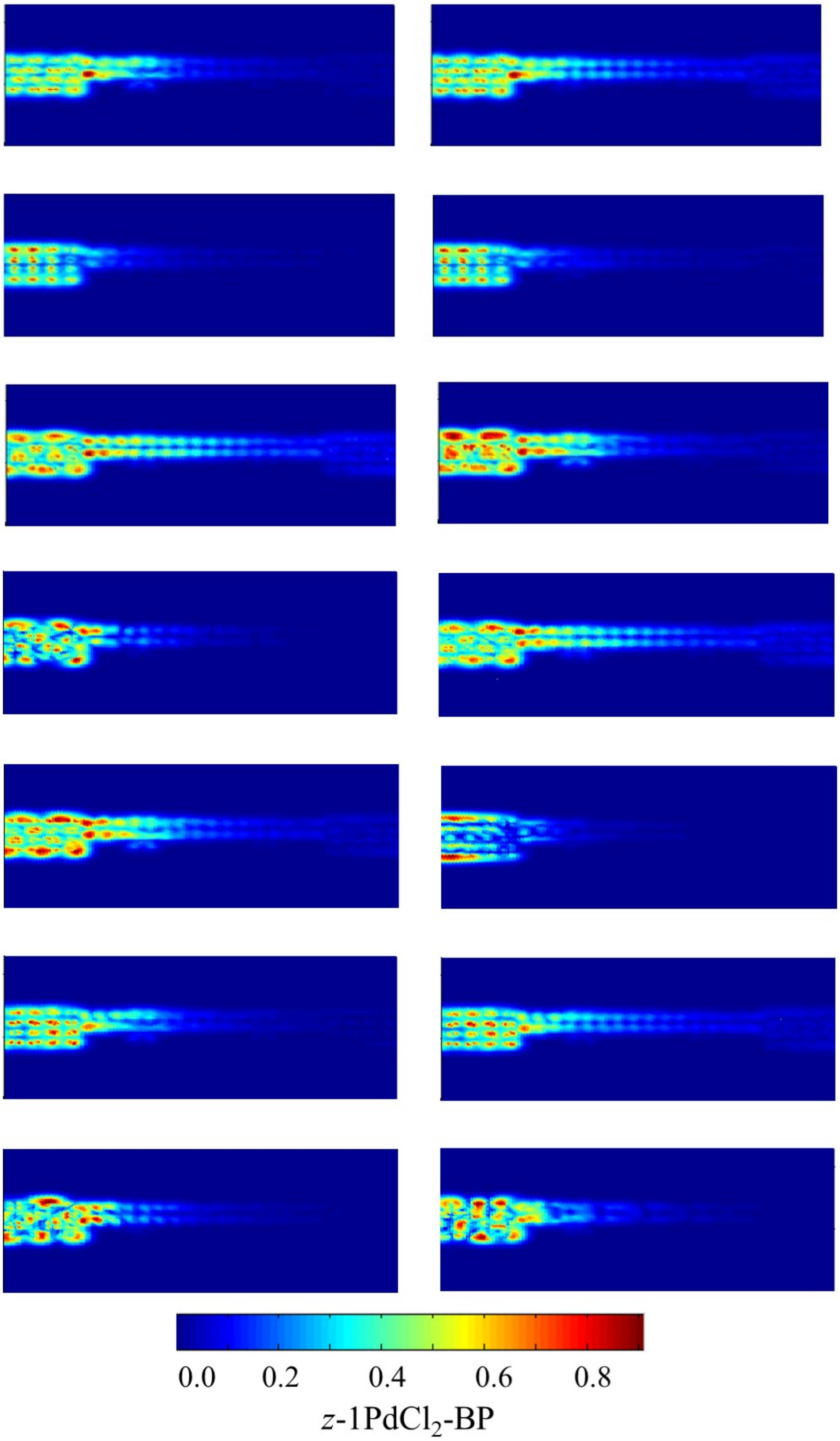

Fig. S6 The real space scattering states of *z*-1$PdCl_2$-BP at Fermi energy E_f.

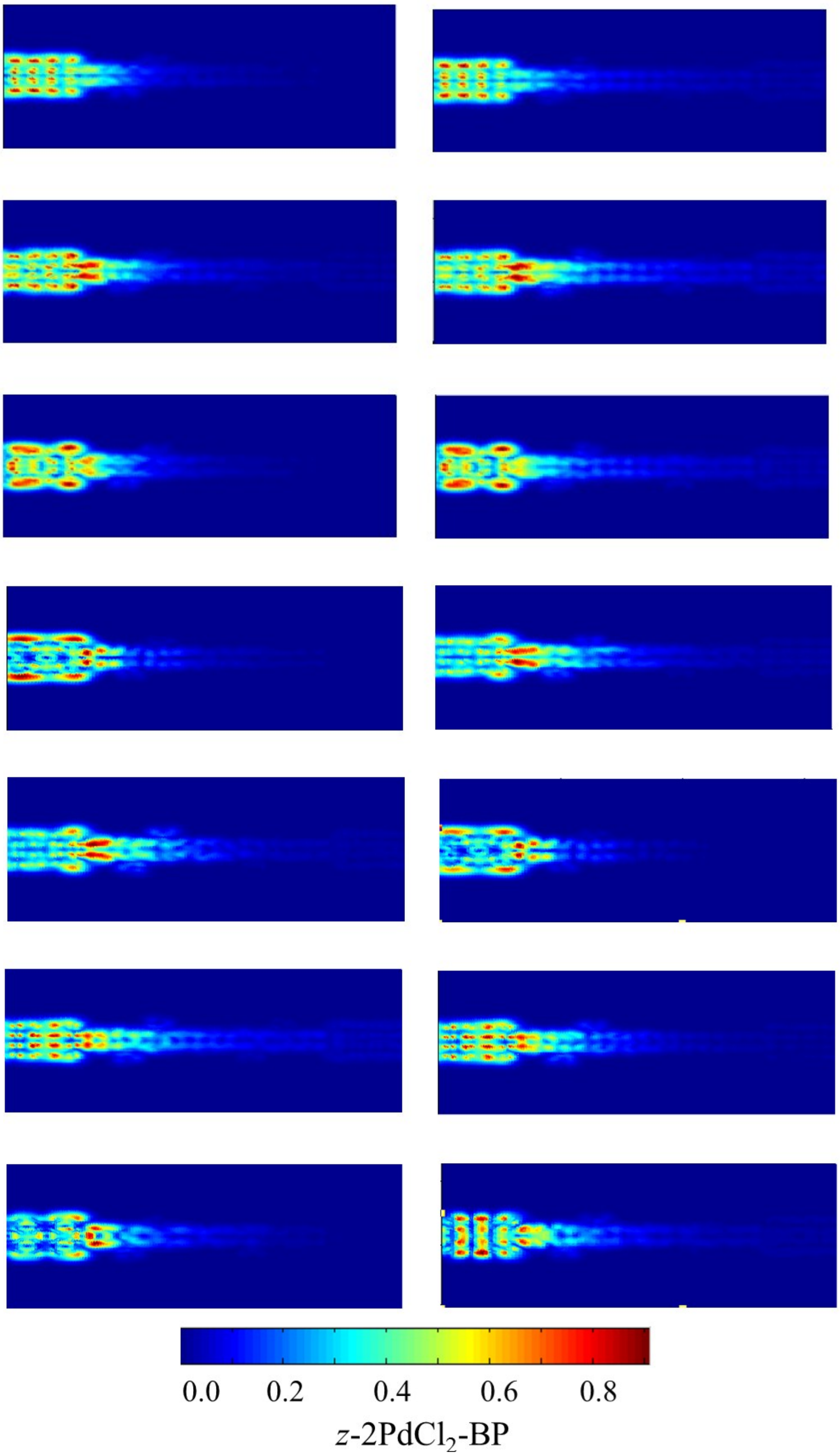

Fig. S7 The real space scattering states of z-2PdCl$_2$-BP at Fermi energy E_f.

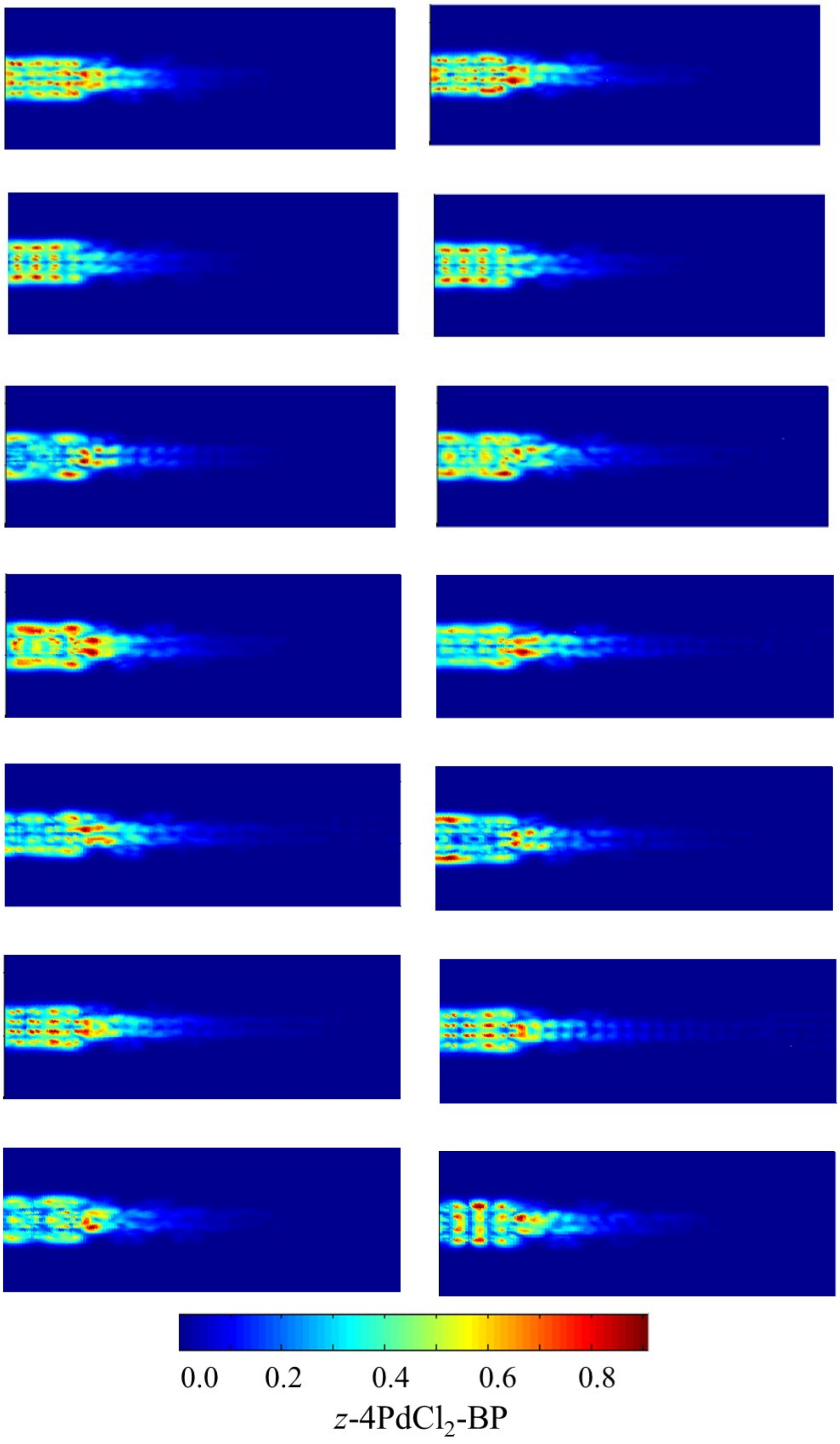

Fig. S8 The real space scattering states of z-4$PdCl_2$-BP at Fermi energy E_f.
